# Preoperative Platelet and International Normalized Ratio Thresholds and Their Impact on Complications Following Revision Total Joint Arthroplasty

**DOI:** 10.1016/j.artd.2026.102119

**Published:** 2026-08-24

**Authors:** Rigel P. Hall, Collin L. Braithwaite, Jens T. Verhey, David G. Deckey, Paul R. Van Schuyver, Ryan Lebens, Joshua S. Bingham, Mark J. Spangehl, Zachary K. Christopher

**Affiliations:** a Department of Orthopaedic Surgery, Creighton University School of Medicine, Phoenix, AZ, USA; b Department of Orthopaedic Surgery, Mayo Clinic, Phoenix, AZ, USA; c Department of Orthopaedic Surgery, Renaissance School of Medicine at Stony Brook University, Stony Brook, NY, USA

**Keywords:** Revision total joint arthroplasty, Platelet count, International normalized ratio, Periprosthetic joint infection, Postoperative complications

## Abstract

**Background:**

Traditional thresholds of platelet counts ≥50,000/μL and international normalized ratio (INR) < 1.5 may not adequately capture the risks of mild coagulopathy. This study evaluated complication rates across varying preoperative platelet and INR values in patients undergoing revision total joint arthroplasty.

**Methods:**

Patients undergoing revision total knee or hip arthroplasties were retrospectively identified from a health care database. Patients were stratified by preoperative platelet counts (50,000 to 100,000/μL, 100,000 to 150,000/μL, >150,000/μL) and INR (<1.0, 1.1 to 1.5, 1.6 to 2.0, >2.0) recorded within 30 days of surgery. Following 1:1 propensity score matching to adjust for confounders, evaluated outcomes included transfusions, bleeding, thromboembolic events, wound complications, periprosthetic joint infection (PJI), reoperation, and mortality up to 2 years.

**Results:**

Platelet counts of 50,000 to 100,000/μL were associated with increased transfusions, greater 90-day bleeding, and higher 2-year PJI and mortality. Counts of 100,000 to 150,000/μL were similarly linked to higher transfusions, PJI, and mortality. An INR of 1.1 to 1.5 correlated with increased 90-day transfusions, wound complications, and thromboembolic events, alongside higher 2-year PJI, reoperation, and mortality. INR levels >1.5 were consistently associated with elevated 90-day thromboembolic events and increased 2-year PJI, with levels of 1.6 to 2.0 also linked to higher mortality.

**Conclusions:**

The findings from this study indicate that preoperative platelet counts below 150,000/μL and INR values above 1.0 were associated with higher rates of complications following revision total joint arthroplasty. These results suggest stricter optimization of hematologic parameters preoperatively may reduce complications.

## Introduction

Relative to 2019 base values, revision total hip arthroplasty (rTHA) is expected to increase by 42% by 2040 and 101% by 2060, while revision total knee arthroplasty (rTKA) is expected to rise by 149% and 520% in those same years, respectively [1]. Infection, instability, and loosening remain the leading indications for revision total joint arthroplasty (rTJA) [2]. Preoperative medical optimization has been shown to reduce surgical risks and mortality following TJA. Several modifiable risk factors, including body mass index, hemoglobin A1c, serum albumin, hemoglobin level, and smoking status have been associated with worse postoperative outcomes following TJA [3].

Platelet count and international normalized ratio (INR) are additional markers used to assess perioperative bleeding risk. Historically, a platelet count ≥50,000/μL and an INR <1.5 have been considered safe thresholds for elective surgery [[4], [5], [6]]. However, recent studies have challenged these cutoffs, demonstrating an increased risk of infection, transfusion, and overall mortality with preoperative INR >1.25 to 1.5 and platelet counts <100,000/μL [7,8]. Importantly, thrombocytopenia and elevated INR may reflect broader systemic frailty and poorer baseline health status rather than only isolated coagulation abnormalities [[9], [10], [11], [12]]. An INR of approximately 1.0 represents normal coagulation, and elevations above this value reflect progressive impairment in clotting function that may contribute to adverse perioperative outcomes [[13], [14], [15], [16]].

To date, there is limited evidence of the impact of preoperative platelet count and INR levels on outcomes following revision THA and TKA [[17], [18], [19]]. Therefore, the purpose of this study was to assess how varying preoperative platelet and INR levels influence the risk of postoperative complications following rTHA and rTKA. We hypothesized that a platelet count <100,000/μL and an INR >1.1 would be associated with an increased risk of postoperative complications after rTJA.

## Material and methods

### Data source

This retrospective study utilized the TriNetX platform (TRINETX LLC, Cambridge, Massachusetts). The database includes the deidentified electronic health records of more than 275 million patients from 127 healthcare organizations. Data on demographic information, medications, diagnoses, and surgical procedures are electronically queried using International Classification of Diseases, tenth revision (ICD-10), Current Procedural Terminology (CPT), and RxNorm codes. This database only includes deidentified data and was deemed exempt by our institutional review board.

### Study population

Patient outcome measures and cohorts were defined using CPT and ICD-10 codes. The date range of this study was June 9, 2005, to June 9, 2025. Treatment cohorts captured within the dataset were patients identified to have undergone rTKA (CPT 27486, 27487) or rTHA (CPT 27134, 27138, 27137) with preoperative platelet counts or INR values within 30 days prior to surgery. When multiple INR or platelet values were available within 30 days of surgery, the value closest to the surgical date was used for analysis to ensure that the laboratory value most representative of the perioperative period was included. Revision of 1 major implant component was required for inclusion, and isolated polyethylene liner exchanges were excluded. Patients were stratified into cohorts based on platelet counts (50,000 to 100,000/μL, 100,000 to 150,000/μL, and >150,000/μL) and INR values (<1.0, 1.1 to 1.5, 1.6 to 2.0, >2.0), consistent with previously published analysis in orthopaedic surgery [20]. Controls were defined as patients with preoperative values of INR <1.0 and platelets >150,000/μL. Propensity scoring was used to match the treatment and control cohorts 1:1 based on age at surgery, sex, obesity, preoperative hemoglobin levels, preoperative albumin levels and Charlson Comorbidity Index. Anticoagulant use—including aspirin, warfarin, heparin products, and direct oral anticoagulants—was included in the matching process. For INR analyses, platelet count was included in the match; for platelet analyses, INR was included. After matching, cohorts were well balanced with the majority of variables demonstrating non-significant standardized differences (Table 1, Table 2).

**Table 1:** Baseline characteristics of patients in platelet count cohorts after propensity score matching.

Characteristics	50,000–100,000/μL (n = 506)		100,000–150,000/μL (n = 2140)	
	Value after PSM	Std. Diff to control	Value after PSM	Std. Diff to control
Age at surgery, y (±SD)	66.6 ± 13.7	0.028	68.8 ± 12.4	0.004
Women, n (%)	241 (47.7)	0.032	1008 (47.0)	0.019
Myocardial infarction, n (%)	66 (13.1)	0.024	240 (11.2)	0.001
Heart failure, n (%)	172 (34.1)	0.013	601 (28.0)	0.023
Peripheral vascular disease, n (%)	65 (12.9)	0.052	241 (11.2)	0.024
Transient ischemic attack, n (%)	47 (9.3)	0.027	195 (9.1)	0.002
Dementia, n (%)	27 (5.3)	0.009	128 (6.0)	0.017
Chronic obstructive pulmonary disease, n (%)	132 (26.1)	0.005	462 (21.5)	0.007
Peptic ulcer disease, n (%)	64 (12.7)	0.128	197 (9.2)	0.038
Diseases of liver, n (%)	145 (28.7)	0.009	382 (17.8)	0.025
Hemiplegia and hemiparesis, n (%)	15 (3.0)	0.064	44 (2.1)	0.007
Body mass index (±SD)	31.0 ± 7.4	0.055	30.8 ± 7.5	0.058
Chronic kidney disease (CKD), n (%)	188 (37.2)	0.025	618 (28.8)	0.009
Human immunodeficiency virus (HIV) disease, n (%)	10 (2.0)	<0.001	24 (1.1)	0.004
Atrial fibrillation and flutter, n (%)	143 (28.3)	0.031	535 (25.0)	0.016
Sepsis, n (%)	140 (27.7)	0.022	395 (18.4)	0.010
Nicotine use, n (%)	115 (22.8)	<0.001	451 (21.0)	0.007
Deep vein thrombosis, n (%)	68 (13.5)	0.045	202 (9.4)	0.011
Pulmonary embolism, n (%)	50 (9.9)	0.013	176 (8.2)	0.003
Transient cerebral ischemic attack, n (%)	34 (6.7)	0.016	130 (6.1)	0.023
Cerebral infarction, n (%)	39 (7.7)	0.022	176 (8.2)	0.010
Preoperative anticoagulant Use, n (%)	445 (88.1)	0.064	1772 (82.6)	0.019
Albumin (±SD)	3.2 ± 0.8	0.495	3.6 ± 0.7	0.152
INR (±SD)	1.2 ± 0.4	0.440	1.1 ± 0.3	0.415
Hemoglobin (±SD)	10.2 ± 2.3	0.426	11.2 ± 2.4	0.170

PSM, propensity score matching; Std, Diff, standard difference; SD, standard deviation.

**Table 2:** Baseline characteristics of patients in INR cohorts after propensity score matching.

Characteristics	INR 1.1-1.5 (n = 14600)		INR 1.6-2.0 (n = 1845)		INR >2.0 (n = 1773)	
	Value after PSM	Std. Diff to control	Value after PSM	Std. Diff to control	Value after PSM	Std. Diff to control
Age at surgery, y (±SD)	65.4 ± 12.0	<0.001	70.1 ± 11.3	0.008	70.5 ± 10.8	0.003
Women, n (%)	8762 (60.0)	0.006	963 (52.1)	0.036	909 (51.3)	0.027
Myocardial infarction, n (%)	724 (5.0)	0.003	210 (11.4)	0.012	198 (11.2)	<0.001
Heart failure, n (%)	1341 (9.2)	0.009	625 (33.8)	0.038	598 (33.7)	0.022
Peripheral vascular disease, n (%)	670 (4.6)	0.005	211 (11.4)	0.028	202 (11.4)	0.027
Transient ischemic attack, n (%)	558 (3.8)	0.019	194 (10.5)	0.012	188 (10.6)	0.019
Dementia, n (%)	387 (2.7)	0.003	101 (5.5)	0.002	81 (4.6)	0.019
Chronic obstructive pulmonary disease, n (%)	1687 (11.6)	0.004	369 (20.0)	0.036	359 (20.2)	0.014
Peptic ulcer disease, n (%)	635 (4.3)	0.008	115 (6.2)	0.004	109 (6.1)	0.030
Diseases of liver, n (%)	1041 (7.1)	0.003	219 (11.9)	0.007	183 (10.3)	0.015
Hemiplegia and hemiparesis, n (%)	119 (0.8)	0.002	36 (1.9)	0.012	30 (1.7)	0.021
Body mass index (±SD)	31.3 ± 7.3	0.023	32.0 ± 7.8	0.174	32.4 ± 7.6	0.193
Chronic kidney disease (CKD), n (%)	1785 (12.2)	0.007	526 (28.5)	0.005	501 (28.3)	0.004
Human immunodeficiency virus (HIV) disease, n (%)	82 (0.6)	0.002	<10 (0.5)^a^	0.014	<10 (0.6)^a^	0.028
Atrial fibrillation and flutter, n (%)	1264 (8.7)	0.002	867 (46.9)	0.018	885 (49.9)	0.011
Sepsis, n (%)	853 (5.8)	0.006	327 (17.7)	<0.001	299 (16.9)	0.023
Nicotine use, n (%)	2363 (16.2)	0.012	316 (17.1)	0.035	263 (14.8)	0.036
Deep vein thrombosis, n (%)	667 (4.6)	0.002	306 (16.6)	0.004	323 (18.2)	0.010
Pulmonary embolism, n (%)	522 (3.6)	0.017	240 (13.0)	0.006	254 (14.3)	0.006
Transient cerebral ischemic attack, n (%)	443 (3.0)	0.008	124 (6.7)	0.006	118 (6.7)	0.007
Cerebral infarction, n (%)	533 (3.7)	0.003	177 (9.6)	0.022	170 (9.6)	0.006
Preoperative anticoagulant use, n (%)	7870 (53.9)	0.007	1684 (91.2)	0.096	1621 (91.4)	0.127
Albumin (±SD)	3.9 ± 0.6	0.085	3.4 ± 0.7	0.408	3.6 ± 0.7	0.201
Platelet count (±SD)	275.1 ± 98.1	0.036	276.1 ± 121.8	0.050	265.5 ± 107.5	<0.001
Hemoglobin (±SD)	12.4 ± 2.1	0.107	10.9 ± 2.2	0.243	11.5 ± 2.2	0.028

PSM, propensity score matching; Std. Diff, standard difference; SD, standard deviation.

a The database does not report counts of ≤10.

### Final cohorts

The control cohort consisted of 53% (7656/14,446) rTKA and 47% (6790/14,446) rTHA. The platelet 100 to 150,000/μL cohort included 50% (2369/4737) rTKA and 50% (2368/4737) rTHA, while the platelet 50 to 100,000/μL cohort included 52% (614/1180) rTKA and 48% (566/1180) rTHA. Among INR cohorts, patients with INR 1.1 to 1.5 included 51% (11,745/23,028) rTKA and 49% (11,283/23,028) rTHA, INR 1.6 to 2.0 included 49% (1098/2241) rTKA and 51% (1143/2241) rTHA, and INR >2.0 included 52% (1131/2176) rTKA and 48% (1045/2176) rTHA.

After propensity score matching, the final platelet cohorts included 506 patients with platelet counts of 50 to 100,000/μL and 2140 patients with counts of 100 to 150,000/μL. The final INR cohorts consisted of 14,600 patients with INR 1.1 to 1.5, 1845 patients with INR 1.6 to 2.0, and 1773 patients with INR >2.0.

### Baseline patient characteristics

Baseline demographic and clinical characteristics, including age, sex, body mass index, and comorbid conditions (eg, cardiovascular disease, chronic kidney disease, liver disease, chronic obstructive pulmonary disease, and nicotine use), were collected for all cohorts. Laboratory parameters such as preoperative albumin, hemoglobin, platelet count, and INR were also compared. After propensity score matching, the majority of variables demonstrated standardized differences below 0.1, indicating adequate covariate balance between groups. Complete demographic and comorbidity data are presented in Tables 1 and 2.

### Study outcomes

The primary outcome was the incidence of a bleeding complication or blood transfusion within 30 or 90 days postoperatively. Blood transfusion was defined as receipt of any blood product transfusion including packed red blood cells, platelets, plasma, or other blood component transfusions. Secondary outcomes included thromboembolic events, emergency department visits, or wound complications within 90 days postoperatively, and periprosthetic joint infection (PJI), reoperation, and mortality within 2 years following surgery.

### Data analyses

Continuous data was presented as either means (± standard deviations) or medians (interquartile ranges). Categorical data were presented as total counts (percentages). Continuous variables were compared using independent *t*-tests for parametric data, while Mann–Whitney *U*-tests were used for nonparametric data. Categorical data were compared using *Chi*-square tests. Patients were propensity score matched using a greedy nearest neighbor matching algorithm with a caliper distance of 0.1 pooled standard deviation of the logit of the propensity score. A standardized difference of <0.1 was used to determine whether cohorts were well matched. Logistic regression was utilized to determine the association between the preoperative platelet and INR values and the subsequent risk of complications. All statistical analyses were performed within the database system. Statistical significance was defined as *P* < .05.

## Results

### Postoperative complications

After propensity score matching, patients with platelet counts of 50,000 to 100,000/μL had significantly increased odds of transfusion within 30 days (odds ratio [OR] 1.81, 95% confidence interval [CI] 1.37 to 2.38) and 90 days (OR 1.88, 95% CI 1.45 to 2.44), as well as higher odds of PJI (OR 1.62, 95% CI 1.26 to 2.07) and mortality (OR 2.59, 95% CI 1.79 to 3.74) at 2 years than controls. Although bleeding complications trended higher at 30 days (OR 1.31, 95% CI 0.99 to 1.74) and 90 days (OR 1.32, 95% CI 1.01 to 1.72), only the 90-day difference reached statistical significance (Table 3).

**Table 3:** Odds of complications following revision total joint arthroplasty at 30 d, 90 d, and 2 y follow-up for patients with a platelet count of 50–100k/μL.

Cohort	Odds ratio (95% CI)	Events +	Events control	*P* value
30 d				
Bleeding complications	1.31 (0.99-1.74)	143/506	117/506	.06
Transfusions	1.81 (1.37-2.38)	178/506	117/506	**<.01**
90 d				
Bleeding complications	1.32 (1.01-1.72)	174/506	144/506	**.04**
Transfusions	1.88 (1.45-2.44)	213/506	141/506	**<.01**
Emergency department visits	1.23 (0.92-1.64)	131/506	112/506	.16
Thromboembolic events	1.44 (0.93-2.23)	53/506	38/506	.10
Wound complications	1.16 (0.77-1.74)	56/506	49/506	.47
2 y				
Periprosthetic joint infection	1.62 (1.26-2.07)	264/506	204/506	**<.01**
Mortality	2.59 (1.79-3.74)	106/506	47/506	**<.01**
Reoperation	0.88 (0.63-1.23)	76/506	85/506	.44

Bolded values indicate statistical significance (P < .05).

Patients with platelet counts of 100,000 to 150,000/μL also demonstrated significantly increased odds of transfusion at 30 days (OR 1.30, 95% CI 1.13 to 1.51) and 90 days (OR 1.27, 95% CI 1.10 to 1.46), and significantly increased odds of PJI (OR 1.30, 95% CI 1.14 to 1.47) and mortality (OR 1.48, 95% CI 1.20 to 1.82) at 2 years. Wound complications also trended higher (OR 1.23, 95% CI 0.99 to 1.53) but did not reach significance (Table 4).

**Table 4:** Odds of complications following revision total joint arthroplasty at 30 d, 90 d, and 2 y follow-up for patients with a platelet count of 100–150k/μL.

Cohort	Odds ratio (95% CI)	Events +	Events control	*P* value
30 d				
Bleeding complications	1.02 (0.88-1.17)	481/2140	475/2140	.83
Transfusions	1.30 (1.13-1.51)	497/2140	403/2140	**<.01**
90 d				
Bleeding complications	0.97 (0.85-1.11)	558/2140	570/2140	.68
Transfusions	1.27 (1.10-1.46)	583/2140	488/2140	**<.01**
Emergency department visits	1.07 (0.92-1.24)	434/2140	412/2140	.40
Thromboembolic events	1.26 (0.99-1.59)	166/2140	134/2140	.06
Wound complications	1.23 (0.99-1.53)	197/2140	163/2140	.06
2 y				
Periprosthetic joint infection	1.30 (1.14-1.47)	855/2140	727/2140	**<.01**
Mortality	1.48 (1.20-1.82)	246/2140	173/2140	**<.01**
Reoperation	1.01 (0.85-1.19)	324/2140	326/2140	.93

Bolded values indicate statistical significance (P < .05).

Among INR cohorts, patients with INR 1.1 to 1.5 had significantly higher odds of transfusion at 30 days (OR 1.15, 95% CI 1.07 to 1.23) and 90 days (OR 1.15, 95% CI 1.08 to 1.23), as well as increased risk of thromboembolic events at 90 days (OR 1.15, 95% CI 1.02 to 1.31), PJI (OR 1.24, 95% CI 1.18 to 1.31), mortality (OR 1.18, 95% CI 1.04 to 1.34), wound complications (OR 1.18, 95% CI 1.06 to 1.30), and reoperation (OR 1.10, 95% CI 1.03 to 1.18) at 2 years (Table 5).

**Table 5:** Odds of complications following revision total joint arthroplasty at 30 d, 90 d, and 2 y follow-up for patients with an INR of 1.1-1.5.

Cohort	Odds ratio (95% CI)	Events +	Events control	*P* value
30 d				
Bleeding complications	1.06 (1.00-1.13)	2418/14600	2299/14600	.06
Transfusions	1.15 (1.07-1.23)	2017/14600	1788/14600	**<.01**
90 d				
Bleeding complications	1.05 (0.99-1.11)	2933/14600	2816/14600	.09
Transfusions	1.15 (1.08-1.23)	2568/14600	2283/14600	**<.01**
Emergency department visits	1.05 (0.98-1.13)	1908/14600	1825/14600	.15
Thromboembolic events	1.15 (1.02-1.31)	538/14600	469/14600	**.03**
Wound complications	1.18 (1.06-1.30)	812/14600	697/14600	**<.01**
2 y				
periprosthetic joint infection	1.24 (1.18-1.31)	3756/14600	3189/14600	**<.01**
Mortality	1.18 (1.04-1.34)	550/14600	469/14600	**<.01**
Reoperation	1.10 (1.03-1.18)	2255/14600	2073/14600	**<.01**

Bolded values indicate statistical significance (P < .05)

Patients with INR 1.6 to 2.0 had significantly increased odds of thromboembolic events at 90 days (OR 1.65, 95% CI 1.33 to 2.05) and higher risk of PJI (OR 1.79, 95% CI 1.57 to 2.04) and mortality (OR 1.39, 95% CI 1.12 to 1.73) at 2 years. Bleeding and transfusion rates were not significantly different from controls (Table 6).

**Table 6:** Odds of complications following revision total joint arthroplasty at 30 d, 90 d, and 2 y follow-up for patients with an INR of 1.6-2.0.

Cohort	Odds ratio (95% CI)	Events +	Events control	*P* value
30 d				
Bleeding complications	1.11 (0.96-1.30)	443/1845	408/1845	.17
Transfusions	1.16 (0.99-1.35)	430/1845	384/1845	.07
90 d				
Bleeding complications	1.13 (0.98-1.31)	544/1845	497/1845	.09
Transfusions	1.12 (0.97-1.30)	501/1845	461/1845	.13
Emergency department visits	1.08 (0.92-1.27)	366/1845	344/1845	.36
Thromboembolic events	1.65 (1.33-2.05)	229/1845	146/1845	**<.01**
Wound complications	0.93 (0.73-1.18)	141/1845	151/1845	.54
2 y				
Periprosthetic joint infection	1.79 (1.57-2.04)	929/1845	668/1845	**<.01**
Mortality	1.39 (1.12-1.73)	217/1845	161/1845	**<.01**
Reoperation	1.09 (0.92-1.30)	316/1845	294/1845	.33

Bolded values indicate statistical significance (P < .05).

Finally, patients with INR >2.0 demonstrated significantly increased odds of thromboembolic events at 90 days (OR 2.00, 95% CI 1.60 to 2.50) and higher risk of PJI at 2 years (OR 1.59, 95% CI 1.38 to 1.83). No other outcomes, including bleeding, transfusion, wound complications, or mortality, reached statistical significance (Table 7).

**Table 7:** Odds of complications following revision total joint arthroplasty at 30 d, 90 d, and 2 y follow up for patients with an INR of >2.0.

Cohort	Odds ratio (95% CI)	Events +	Events control	*P* value
30 d				
Bleeding complications	1.10 (0.94-1.29)	419/1773	389/1773	.23
Transfusions	1.01 (0.89-1.15)	381/1773	377/1773	.87
90 d				
Bleeding complications	1.10 (0.94-1.27)	497/1773	472/1773	.34
Transfusions	1.05 (0.91-1.21)	450/1773	426/1773	.35
Emergency department visits	1.00 (0.87-1.15)	334/1773	334/1773	1.00
Thromboembolic events	2.00 (1.60-2.50)	249/1773	134/1773	**<.01**
Wound complications	0.95 (0.75-1.22)	136/1773	142/1773	.71
2 y				
Periprosthetic joint infection	1.59 (1.38-1.83)	724/1773	538/1773	**<.01**
Mortality	1.40 (0.96-2.00)	65/1773	47/1773	.08
Reoperation	0.93 (0.71-1.22)	111/1773	119/1773	.59

Bolded values indicate statistical significance (P < .05).

## Discussion

In the present study, lower preoperative platelet counts and moderately elevated INR values were associated with higher transfusion rates and long-term complications following rTJA. Moderate thrombocytopenia correlated with increased incidence of postoperative transfusion, while more severe reductions were linked to both short-term complications and 2-year mortality. INR elevations were associated with higher rates of thromboembolic events, infections, and increased risk of revision; however, these risks did not demonstrate a stepwise increase at higher INR levels.

The optimal platelet count and INR thresholds for safely performing orthopaedic surgery remain a topic of ongoing debate, with limited evidence available supporting the general consensus platelet threshold of 50,000/μL and INR of <1.5 [[4], [5], [6]]. The majority of the literature available focuses on nonorthopaedic procedures and has varied results [16,21]. Recent orthopaedic literature has challenged these traditional thresholds. Chung et al. [18] established that abnormal coagulation profiles are associated with adverse outcomes across all total joint arthroplasty procedures. Varady et al. [20] found that in operative treatment for hip fracture, patients below a more conservative platelet count cutoff of 100,000/μL were at increased risk of complications, while Malpani et al. [22] identified patients below the threshold of 116,000/μL to be at increased risk of adverse events and greater length of stay following TKA. Rudasill et al. found the risks of postoperative bleeding, infection, and mortality risks in elective total knee and hip arthroplasty to increase in a stepwise manner with higher preoperative INR, with significant risk emerging above 1.25 [8,23]. Bujnowski et al. [7] found thrombocytopenia with levels below 150,000/μL was associated with increased rates of blood transfusion, but not with increased length of hospital stay or 30-day readmission. Together, these findings suggest that even mild coagulopathy may carry clinical significance—an effect further examined in our revision cohort.

Specifically, in revision arthroplasty, greater INR values have been indicated to be a risk factor for complications [18]. Churchill et al. [17] reported that even modest INR elevations (>1.03) in rTKA were independently associated with increased risks of mortality, bleeding requiring transfusion, infection, organ dysfunction, and prolonged hospital stay. Similarly, Arnold et al. [19], using a large national database, found a stepwise increase in adverse outcomes—including mortality, bleeding, sepsis, stroke, ventilator dependence, readmission, and prolonged length of stay—in rTHA, with risk rising at INR values >1.25, suggesting that current INR safety thresholds may warrant re-evaluation in revision arthroplasty populations. Consistent with these studies, our findings support that even mild deviations in coagulation parameters were associated with increased perioperative risk in revision arthroplasty, underscoring the need for tighter preoperative optimization.

Various mechanisms contribute to the increased complications associated with abnormal preoperative platelet counts and INR values. The most direct mechanism is impaired coagulation, which disrupts the normal hemostatic cascade and reduces the formation of stable fibrin clots, leading to increased intraoperative and postoperative bleeding [[24], [25], [26], [27], [28]]. Thrombocytopenia limits platelet plug formation, while elevated INR primarily reflects reduced levels or function of vitamin K–dependent clotting factors or more broadly, impaired hepatic synthesis or consumption of clotting factors, prolonging coagulation time [[29], [30], [31], [32]]. In addition to their role in coagulation physiology, abnormal platelet counts and elevated INR values may function as indicators of overall patient comorbidity and physiologic reserve, which may further contribute to the increased complication risk observed in these patients [[9], [10], [11], [12]]. Perioperative bleeding events often necessitate transfusion—a treatment itself linked to higher morbidity and mortality in orthopaedic surgery [7,[33], [34], [35], [36]]. This highlights the clinical dilemma of managing moderate thrombocytopenia, where the risks of platelet transfusion must be balanced against untreated coagulopathy.

Beyond transfusion, optimization strategies for patients with abnormal coagulation parameters include identifying and correcting reversible causes. In thrombocytopenic patients, assessment of nutritional deficiencies (iron, folate, and vitamin B12), medication-induced suppression, or hepatic dysfunction may allow targeted correction before surgery [37]. Among anticoagulated patients, reversal protocols using vitamin K, prothrombin complex concentrate, or fresh frozen plasma are well established and may explain the attenuation of risk observed at higher INR values [6,[37], [38], [39], [40], [41]]. Incorporating these measures into preoperative optimization may help reduce bleeding and infectious complications in revision arthroplasty. For warfarin-treated patients, reversal is guided by procedure urgency and INR level: warfarin is typically held for 5 days before elective surgery, with low-dose vitamin K considered if INR remains >1.5 [6,42]. In urgent or emergent cases, rapid reversal is achieved with intravenous vitamin K and four-factor prothrombin complex concentrate, which restores vitamin K–dependent clotting factors [[38], [39], [40], [41], [42]]. Guideline-supported strategies likely contribute to the reduced complication risk observed in patients with markedly elevated INRs.

Elevated INR has also been independently associated with wound complications, likely secondary to hematoma formation that impairs wound healing and creates a favorable environment for bacterial colonization [23,43]. Beyond immediate surgical blood loss, persistent wound drainage, hematoma formation, and compromised hemostasis are well-established predictors of postoperative infection after total joint replacement [[44], [45], [46], [47], [48]]. Consistently, patients anticoagulated with warfarin demonstrate increased risks of deep infection, wound hematoma, and superficial infection following total hip arthroplasty, and coagulopathy itself has been identified as an independent risk factor for PJI [[49], [50], [51], [52]]. Collectively, these findings reinforce that abnormal coagulation parameters not only increase bleeding complications but also impair wound healing and raise infection risk in arthroplasty.

This study expands on previous studies by analyzing both safe platelet and INR thresholds specifically within a revision arthroplasty population, and our findings corroborate previous findings that the optimal platelet counts and INR values for orthopaedic surgery may be greater and lower, respectively, than previously thought.

However, unlike previous literature [8,17,19,20,23], this study did not demonstrate a stepwise increase in complication risk with progressively higher preoperative INR values. While the 1.1 to 1.5 cohort showed increased risk for transfusions, thromboembolic events, PJI, and reoperation, the 1.6 to 2.0 cohort was associated with increased thromboembolic events, PJI, and mortality, and the >2.0 cohort was associated with increased thromboembolic events and PJI only. Several factors may explain this discrepancy. The patient counts after matching for the 1.6 to 2.0 (n = 1845) and >2.0 (n = 1773) cohorts were notably smaller than the 1.1 to 1.5 cohort (n = 14,600), limiting statistical power to detect additional associations. Moreover, perioperative optimization or reversal strategies may have mitigated complication risks in patients with more markedly elevated INR values. Although patients were matched for preoperative anticoagulation therapy and hemoglobin levels, differences in postoperative management for those with significantly elevated INRs could have influenced complication rates [53]. Finally, institutional variation in defining or reporting bleeding complications may have further affected the observed associations.

Although elevated INR was associated with increased transfusion rates, no corresponding increase in coded bleeding complications was observed, likely reflecting greater bleed severity rather than frequency and highlighting the limitations of administrative coding. This discrepancy may also reflect unrecorded intraoperative bleeding, which would not be captured by postoperative diagnosis codes. The ICD-10 codes used to define postoperative hemorrhage primarily describe the anatomical site or timing of bleeding rather than its magnitude, limiting sensitivity for distinguishing minor from clinically significant events. Therefore, our findings likely reflect differences in bleed severity rather than incidence, underscoring the limitations of administrative coding in capturing nuanced perioperative outcomes.

In addition, the observed increase in thromboembolic events among patients with INR values between 1.6 to 2.0 and >2.0 may reflect confounding. These patients are often chronically anticoagulated due to conditions such as atrial fibrillation or prior venous thromboembolism and at baseline have a higher thrombotic risk. Perioperative reversal or temporary interruption of warfarin, variability in bridging strategies, and delayed postoperative re-initiation can transiently shift the hemostatic balance toward thrombosis. These variables, which due to the nature of the study could not be captured, likely contributed to this counterintuitive association and represent a limitation of this analysis.

### Limitations

The findings of this study should be interpreted in the context of several limitations. This was a database study that relied on the accuracy of ICD and CPT codes when examining the occurrence of complications after surgery and therefore is susceptible to electronic health record coding errors and underreporting of potential complications. However, the accuracy of ICD-10 and CPT coding in total joint arthroplasty research has been validated through comparison with chart reviews [54]. The database also enhances data capture by incorporating natural language processing algorithms alongside traditional coding methods [55]. The natural language processing–based data extraction in total joint arthroplasty research has been demonstrated to be a reliable approach, further supporting the accuracy of the database and addressing concerns regarding data integrity [54,56].

Next, several intraoperative and perioperative variables could not be reliably assessed. Operative time, estimated intraoperative blood loss, use of tranexamic acid, tourniquet application, drain utilization, negative pressure wound therapy, and perioperative antibiotic protocols are not consistently recorded in the database and therefore could not be included in the analysis. Each of these factors may influence bleeding, transfusion requirements, wound complications, or infection risk and represent sources of residual confounding. Baseline anemia may also represent a potential confounder in this analysis. Patients in several cohorts demonstrated relatively low preoperative hemoglobin levels, which may independently increase the likelihood of perioperative transfusion and adverse postoperative outcomes. Although preoperative hemoglobin was included in the propensity score matching process, residual confounding related to underlying anemia and medical frailty may persist.

Although rTJA encompasses a broad spectrum of procedural complexity, this analysis was limited to component-level revision procedures. Isolated polyethylene liner exchanges were excluded, and revision of at least 1 major implant component was required for inclusion, thereby restricting the cohort to moderate-to-high complexity revision cases. In addition, both revision total hip and knee arthroplasties were included in this analysis to evaluate broader revision arthroplasty populations; however, these procedures differ in operative characteristics and complication profiles. Despite propensity score matching, variability between single-component and multicomponent revisions as well as between hip and knee procedures may influence operative duration, blood loss, and complication risk.

Additionally, the sample size was smaller than typical in database studies, which was expected given that most patients meet recommended platelet and INR thresholds different from those used as inclusion criteria in this analysis. Furthermore, due to the nature of the dataset, we could not account for postoperative anticoagulation practices or interventions beyond transfusion. The database lacks detail on surgical technique, implant type, or hospital volume, which are other potential confounders. Variation in defining or reporting bleeding complications may have further affected outcome reporting in cases such as a hematoma determined to be subclinical. As previously discussed, the codes used to identify postoperative hemorrhage capture anatomical location and timing but not severity, which may underestimate clinically meaningful bleeding events. It must also be acknowledged that events occurring at the 2-year follow-up may not necessarily be directly attributable to the index surgery itself, and database constraints limit confirmation that identified PJI events involved the same revised joint. Nonetheless, inclusion of these outcomes provides valuable insight into broader postoperative risk patterns within large population-level datasets.

This analysis spans a 20-year period during which substantial changes in rTJA practice have occurred. Transfusion strategies have become increasingly restrictive, with modern clinical practice adopting lower hemoglobin thresholds for transfusion, reducing transfusion exposure [57]. In addition, strategies to reduce intraoperative bleeding such as permissive hypotension have become increasingly utilized [58,59]. Preoperative autologous donation has largely fallen out of favor due to poor cost-effectiveness, advances in blood conservation techniques, and reduction of infection risk associated with allogenic transfusions [58,60]. These developments, combined with the widespread adoption of tranexamic acid, reflect evolving surgical and perioperative management strategies over time [[61], [62], [63]]. Another important shift is that most patients in the United States are no longer managed with warfarin postoperatively. Many arthroplasty surgeons now utilize aspirin for postoperative thromboprophylaxis due to elimination of routine INR monitoring, reduced wound drainage, demonstration of effectiveness compared with other anticoagulant medications in patients with significant comorbidities, and association with reduced mortality following primary arthroplasty [51,[64], [65], [66], [67]]. However, this study may be particularly relevant for surgeons practicing in settings outside the United States, where warfarin use remains more common. These temporal shifts may independently influence complication rates, introducing potential confounding that cannot be fully accounted for in a retrospective database analysis.

Despite these limitations, the findings align with prior large registry studies [8,[17], [18], [19], [20],^23^], supporting their validity and reinforcing the need to reconsider widely accepted hemostatic thresholds for revision arthroplasty.

## Conclusions

This matched, retrospective cohort analysis evaluated the impact of preoperative platelet count and INR thresholds on postoperative complications following rTJA. The findings of this study corroborate prior evidence that even moderate coagulopathy can be associated with an increased perioperative risk and suggest that platelet counts below 150,000/μL and INR values above 1.0 are associated with adverse outcomes. These results suggest that stricter preoperative optimization of hematologic parameters may reduce complication rates in the revision arthroplasty population. However, it is important to recognize that not all patients can achieve corrected values due to underlying pathology. In such cases, understanding these associated risks enables more informed patient counseling regarding risks and benefits and underscores the importance of individualized perioperative management for patients with even mild coagulopathy.

## Conflict of interest

David G. Deckey reports stock or stock options in Osgenic and Sonoran Biosciences; editorial or governing board roles with *The Journal of Arthroplasty* and *The Journal of Knee Surgery*; committee or board appointments with AAHKS and the Knee Society. Mark Spangehl reports research support as a principal investigator from Stryker and DePuy Synthes; editorial or governing board roles with *The Journal of Arthroplasty*; and committee or board appointments with the Knee Society. All other authors declare no potential conflicts of interest.

For full disclosure statements refer to https://doi.org/10.1016/j.artd.2026.102119.

## CRediT authorship contribution statement

**Rigel P. Hall:** Writing – review & editing, Writing – original draft, Investigation, Formal analysis, Data curation, Conceptualization. **Collin L. Braithwaite:** Writing – review & editing, Writing – original draft, Investigation, Formal analysis, Data curation, Conceptualization. **Jens T. Verhey:** Writing – review & editing, Writing – original draft, Visualization, Supervision, Methodology, Investigation, Conceptualization. **David G. Deckey:** Writing – review & editing, Visualization, Supervision, Methodology, Investigation, Conceptualization. **Paul R. Van Schuyver:** Writing – review & editing, Supervision, Methodology, Investigation. **Ryan Lebens:** Writing – review & editing, Software, Methodology, Investigation, Formal analysis, Data curation. **Joshua S. Bingham:** Writing – review & editing, Visualization, Supervision, Conceptualization. **Mark J. Spangehl:** Writing – review & editing, Visualization, Supervision, Conceptualization. **Zachary K. Christopher:** Writing – review & editing, Supervision, Methodology, Investigation, Conceptualization.

## Appendix A Supplementary data

Conflict of Interest Statement for Bingham

Conflict of Interest Statement for Braithwaite

Conflict of Interest Statement for Deckey

Conflict of Interest Statement for Verhey

Conflict of Interest Statement for Van Schuyver

Conflict of Interest Statement for Lebens

Conflict of Interest Statement for Hall

Conflict of Interest Statement for Spangehl

Conflict of Interest Statement for Christopher
